# Marginal turbid band and light blue crest, signs observed in magnifying narrow-band imaging endoscopy, are indicative of gastric intestinal metaplasia

**DOI:** 10.1186/1471-230X-12-169

**Published:** 2012-11-27

**Authors:** Jin Kwang An, Geun Am Song, Gwang Ha Kim, Do Youn Park, Na Ri Shin, Bong Eun Lee, Hyun Young Woo, Dong Yup Ryu, Dong Uk Kim, Jeong Heo

**Affiliations:** 1 Department of Internal Medicine, Pusan National University School of Medicine, and Biomedical Research Institute, Pusan National University Hospital, 1-10, Ami-dong, Seo-gu, Busan, 602-739, Korea; 2 Department of Pathology, Pusan National University School of Medicine, Busan, Korea

**Keywords:** Stomach, Magnifying endoscopy, Intestinal metaplasia

## Abstract

**Background:**

Gastric intestinal metaplasia (IM) usually appears in flat mucosa and shows few morphologic changes, making diagnosis using conventional endoscopy unreliable. Magnifying narrow-band imaging (NBI) endoscopy enables evaluation of detailed morphological features that correspond with the underlying histology. The aim of this study was to investigate and clarify the diagnostic efficacy of magnifying NBI endoscopic findings for the prediction and diagnosis of IM.

**Methods:**

Forty-seven patients were prospectively enrolled, and magnifying NBI examinations were performed in the lesser curvature of the midbody and the greater curvature of the upper body. The marginal turbid band (MTB) was defined as an enclosing white turbid band on the epithelial surface/gyri; light blue crest (LBC), as a fine, blue-white line on the crest of the epithelial surface/gyri. Immediately after observation under magnifying endoscopy, biopsy specimens were obtained from the evaluated areas.

**Results:**

The degree of IM significantly increased with increasing MTB/LBC positivity (MTB^-^/LBC^-^, 0.00 ± 0.00; MTB^+^/LBC^-^, 0.44 ± 0.51; MTB^+^/LBC^+^, 0.94 ± 0.24; *p* < 0.001). Moderate-to-severe IM was more common in MTB^+^/LBC^+^ areas than in MTB^+^/LBC^-^ areas (*p* < 0.001). For the diagnosis of IM, MTB had a sensitivity, specificity, and accuracy of 100%, 66.0%, and 81.7%, respectively, and the corresponding values for LBC were 72.1%, 96.0%, and 84.9%.

**Conclusion:**

MTB and LBC observed in the gastric mucosa with magnifying NBI endoscopy are highly accurate indicators of the presence of IM. MTB likely represents a sign of early gastric IM, while LBC appears with progression to severe IM.

## Background

Gastric intestinal metaplasia (IM) is regarded as a precancerous lesion that is likely to develop into intestinal-type gastric cancer [1]. Hence, endoscopic diagnosis of IM is valuable for patients undergoing surveillance endoscopy [2]. The diagnosis of IM is currently based on the histological assessment of biopsy specimens. Diagnosis of IM using conventional endoscopy is unreliable because IM usually appears in flat mucosa and shows few macroscopic morphological changes [3-5]. In addition, because of the large surface area of the stomach, only small areas can be sampled with random biopsies [6]. IM can be focal and may be missed on random biopsies. Multiple non-targeted biopsies also add to the cost and the time it takes to perform the procedure, without necessarily improving the diagnostic yield.

Recent techniques that allow high-resolution visualization of mucosal details may help bring the focus on endoscopic examination of the stomach, and aid in cost-effective and time-efficient disease diagnosis. Narrow-band imaging (NBI) is an endoscopic imaging technology that uses blue (400–430 nm) and green (535–565 nm) narrow-band, short-wavelength light to improve the contrast of surface structures and vascular architecture in the superficial mucosa. Magnifying NBI endoscopy enables evaluation of detailed morphological features of the epithelium corresponding to histological findings [7-9]. For example, a recent study reported that the appearance of a light blue crest (LBC) in the mucosa is a distinctive endoscopic finding that suggests an increased probability of IM [10].

However, only a few studies have provided additional details regarding the clinical significance and reproducibility of using LBC as a magnifying NBI endoscopic finding for predicting IM [11,12]. The aim of this study was to investigate magnifying NBI endoscopic findings for the prediction of IM and to clarify the diagnostic efficacy of these findings for the detection of IM.

## Methods

### Study population

Forty-seven patients (24 men and 23 women), with a mean age of 55 years (range, 23–68 years) were prospectively enrolled from September 2009 to April 2010. Of them, 15 visited our hospital primarily for treatment of early gastric cancer. Other patients underwent upper endoscopy for various other indications, including yearly screening for gastric cancer and complaints of abdominal discomfort or dyspepsia. Patients with severe systemic diseases or advanced chronic liver diseases; those receiving H_2_ receptor antagonists, proton-pump inhibitors, or non-steroidal anti-inflammatory drugs; those who had received *Helicobacter pylori* (*H*. *pylori*) eradication therapy; and those with a history of gastric surgery were excluded from the study. This study was reviewed and approved by the Institutional Review Board at Pusan National University Hospital. Written informed consent was obtained from all patients.

### Endoscopic procedures

The video endoscopy system used was the EVIS-LUCERA SPECTRUM system (Olympus Medical Systems Corp., Tokyo, Japan), which consisted of a light source (CLV-260SL), a processor (CV-260SL), and a magnifying video endoscope (GIF-H260Z). The system was capable of both white light and NBI modes, which could be toggled within 1 minute using a button on the control head of the video endoscope. This endoscopy system has a zoom magnification of 80×. To obtain a clear view with magnifying endoscopy, a transparent hood, MB-46 (Olympus), was fitted on the distal tip of the endoscope to maintain the focal distance.

**Figure 1:**
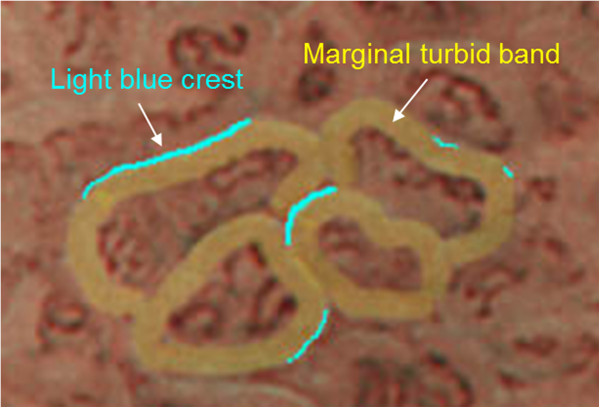
**Schematic figure for marginal turbid band and light blue crest.** The marginal turbid band is defined as an enclosing, white turbid band on the epithelial surface/gyri, and light blue crest is defined as a fine, blue-white line on the crest of the epithelial surface/gyri.

**Figure 2:**
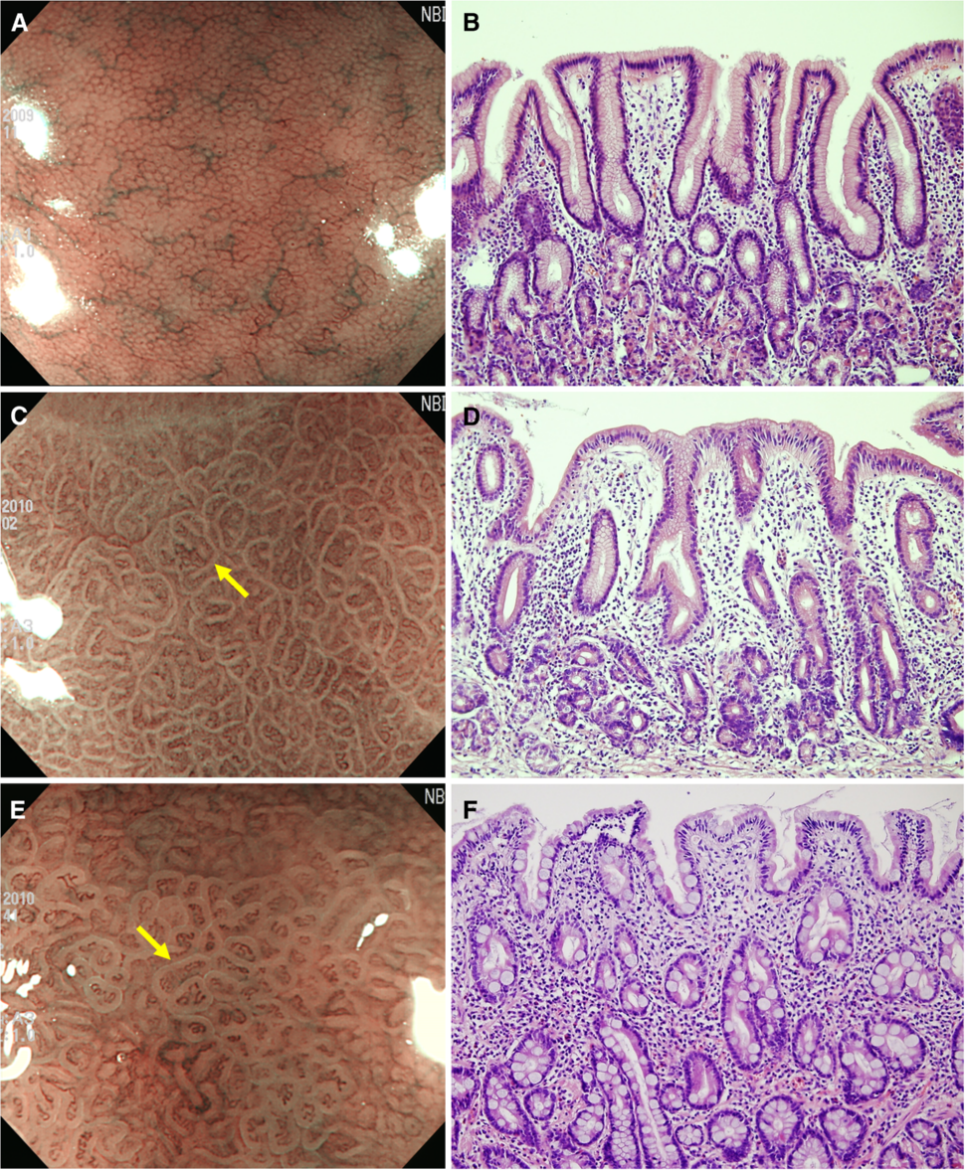
**Magnifying NBI endoscopic findings and representative histological findings. A** Uniform round pits surrounded by a regular honeycomb subepithelial network and a regular arrangement of collecting venules are seen. No marginal turbid band (MTB) or light blue crest (LBC) is observed. **B** Histological view showing no atrophy or intestinal metaplasia. **C** Regular honeycomb subepithelial network and collecting venules subside. MTBs are seen (*arrow*), but LBC is shadowy. **D** Histological view illustrating a mild degree of intestinal metaplasia and atrophy. **E** Both MTBs are LBCs are distinctly visible (*arrow*). **F** Histological view showing moderate to severe mucosal atrophy and intestinal metaplasia.

The preparation procedure for the magnifying endoscopic examination was the same as that for conventional endoscopy. After topical anesthesia, the scope was inserted into the stomach, and routine observation was performed. Next, magnifying NBI examinations of 2 areas of the stomach were performed: an area located at the lesser curvature of the midbody and another area at the greater curvature of the upper body. The marginal turbid band (MTB) was defined as an enclosing, white turbid band on the epithelial surface/gyri, and LBC was defined as a fine, blue-white line on the crest of the epithelial surface/gyri (Figures 1, 2) [10]. A positive MTB or LBC was defined as MTB or LBC >10%. All LBC-positive areas were also MTB-positive, allowing classification of the areas into 3 groups: MTB^-^/LBC^-^, MTB^+^/LBC^-^, and MTB^+^/LBC^+^. Immediately after observation under magnifying endoscopy, 1 biopsy specimen was obtained from each evaluated area. All endoscopic procedures were carried out by a single endoscopist (G.H. Kim) with previous experience in magnifying endoscopy.

### Histological assessment

Biopsy specimens were fixed using buffered formalin and then embedded in paraffin. An expert pathologist (D.Y. Park) who was blinded to the endoscopic findings examined all histological samples. Histological variables, including *H*. *pylori*, neutrophil infiltration (activity), mononuclear cell infiltration (inflammation), atrophy, and IM were graded according to a visual analog scale in the updated Sydney system (e.g., none (0), mild (1), moderate (2), and severe (3)) [13].

### Statistical analysis

Data are expressed as mean ± SD. Differences in histological variables associated with the presence of MTB and LBC were assessed using the Student’s *t*-test. Differences in histological variables among the 3 groups (MTB^-^/LBC^-^, MTB^+^/LBC^-^, and MTB^+^/LBC^+^) were assessed using the one-way analysis of variance (ANOVA) test. A χ^2^ test was performed to assess the differences in the grade of atrophy and IM among the 3 groups. Calculations of the sensitivity, specificity, and the positive and negative predictive values of MTB and LBC for predicting atrophy and IM were also performed. A *p*-value <0.05 was considered statistically significant. Statistical calculations were performed using SPSS version 12.0 for Windows software (SPSS Inc., Chicago, IL, USA).

## Results

### Histological findings

Of the 94 biopsy specimens, 1 specimen obtained from the midbody was insufficient for histological analysis. As a result, 93 areas (46 in the lesser curvature of the midbody and 47 in the greater curvature of the upper body) were included in this study. Sixty of the 93 areas (39 in the midbody and 21 in the upper body) were MTB positive, of which 55 (91.7%) and 43 (71.7%) showed histological evidence of atrophy and IM, respectively. Thirty-three areas (22 in the midbody and 11 in the upper body) were LBC positive, of which 32 (97.0%) and 31 (93.9%) showed histological evidence of atrophy and IM, respectively. MTB was also positive in all 33 areas that were LBC positive. MTB-positive areas showed a higher degree of inflammation, atrophy, and IM than did MTB-negative areas (Table 1). LBC-positive areas showed a lower density of *H*. *pylori* and a higher degree of atrophy and IM than did LBC-negative areas.

**Table 1:** Presence or absence of the marginal turbid band or light blue crest and association with histological variables

**Histological variables**	**Marginal turbid band**		***p-*value**	**Light blue crest**		***p-*value**
	**Absent (n = 33)**	**Present (n = 60)**		**Absent (n = 60)**	**Present (n = 33)**	
*Helicobacter pylori*	0.58 ± 0.79	0.47 ± 0.68	0.485	0.62 ± 0.76	0.30 ± 0.59	0.030
Acute inflammation	0.42 ± 0.61	0.45 ± 0.59	0.844	0.43 ± 0.62	0.45 ± 0.56	0.871
Chronic inflammation	1.24 ± 0.44	1.47 ± 0.50	0.028	1.38 ± 0.49	1.39 ± 0.50	0.921
Atrophy	0.45 ± 0.56	1.00 ± 0.41	<0.001	0.63 ± 0.52	1.12 ± 0.42	<0.001
Intestinal metaplasia	0.00 ± 0.00	1.23 ± 0.98	<0.001	0.23 ± 0.50	1.82 ± 0.81	<0.001

**Figure 3:**
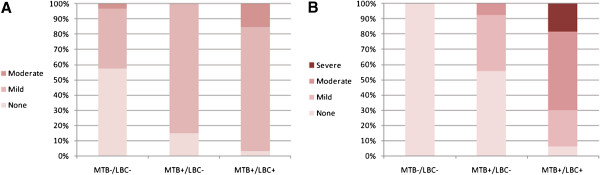
**A, B The relationship between magnifying NBI endoscopic findings and histological findings.** There were significant differences in the grades of atrophy (*p* < 0.001) and intestinal metaplasia (*p* < 0.001) among the 3 groups categorized by the presence of marginal turbid band (MTB) and light blue crest (LBC).

When groups classified according to the presence or absence of MTB and LBC were compared, the degree of atrophy was significantly higher in the MTB^+^/LBC^-^ and MTB^+^/LBC^+^ groups than the MTB^-^/LBC^-^ group (0.85 ± 0.36, 1.12 ± 0.42, and 0.45 ± 0.56, respectively, *p* < 0.001) (Table 2). The degree of IM significantly increased with increasing MTB/LBC positivity (MTB^-^/LBC^-^, 0.00 ± 0.00; MTB^+^/LBC^-^, 0.44 ± 0.51; MTB^+^/LBC^+^, 0.94 ± 0.24; *p* < 0.001). Moderate-to-severe IM was more commonly seen in MTB^+^/LBC^+^ areas than in MTB^+^/LBC^-^ areas (*p* < 0.001) (Figure 3).

**Table 2:** Marginal turbid band (MTB) and light blue crest (LBC) categories and association with histological variables

**Histological variables**	**MTB**^**-**^**/LBC**^**-**^	**MTB**^**+**^**/LBC**^**-**^	**MTB**^**+**^**/LBC**^**+**^	*p-*value*
	**(n = 33)**	**(n = 27)**	**(n = 33)**	
*Helicobacter pylori*	0.58 ± 0.79	0.67 ± 0.73	0.30 ± 0.59	0.115
Acute inflammation	0.42 ± 0.61	0.44 ± 0.64	0.45 ± 0.56	0.979
Chronic inflammation	1.24 ± 0.44	1.56 ± 0.51	1.39 ± 0.50	0.046
T^†^	a	b	a,b	
Atrophy	0.45 ± 0.56	0.85 ± 0.36	1.12 ± 0.42	<0.001
T^†^	a	b	b	
Intestinal metaplasia	0.00 ± 0.00	0.44 ± 0.51	0.94 ± 0.24	<0.001
T^†^	a	b	c	

* Statistical significances were tested using one-way ANOVA.

^†^ The same letters indicate non-significant difference between groups on Tukey’s multiple comparison test.

### Accuracy of MTB and LBC for diagnosis of atrophy and IM

For the diagnosis of atrophy, MTB had a sensitivity, specificity, and accuracy of 79.7%, 79.2%, and 79.6%, respectively, and the corresponding values for LBC were 46.4%, 95.8%, and 59.1 (Table 3). For the diagnosis of IM, MTB had a sensitivity, specificity and accuracy of 100%, 66.0%, and 81.7%, respectively, and the corresponding values for LBC were 72.1%, 96.0%, and 84.9%.

**Table 3:** Sensitivity, specificity, positive and negative predictive values, and accuracy of magnifying NBI endoscopic findings for predicting gastric atrophy and intestinal metaplasia

	**Sensitivity (%)**	**Specificity (%)**	**PPV (%)**	**NPV (%)**	**Accuracy (%)**
Prediction of atrophy					
Marginal turbid band	79.7	79.2	91.7	57.6	79.6
Light blue crest	46.4	95.8	97.0	38.3	59.1
Prediction of intestinal metaplasia					
Marginal turbid band	100	66.0	71.7	100	81.7
Light blue crest	72.1	96.0	93.9	80.0	84.9

PPV, positive predictive value; NPV, negative predictive value.

## Discussion

In this study, magnifying NBI endoscopy was used to classify gastric epithelium on the basis of the presence or absence of MTB/LBC. Our results suggest an association between histological findings on gastric biopsy and areas positive for MTB and/or LBC. Areas positive for MTB or LBC were associated with atrophy and IM. In addition, MTB/LBC positivity was associated with the severity of IM, such that the grade of IM in the MTB^+^/LBC^+^ group was more severe than that in the MTB^+^/LBC^-^ group.

Many studies have investigated the use of magnifying endoscopy for overcoming the diagnostic limitations of IM with conventional endoscopy [10]. Magnifying endoscopy with methylene blue staining has been reported to be useful in the diagnosis of IM (sensitivity, 76.4%; specificity, 86.6%) [14]. However, the limitations associated with this method include the need for preparation with mucolytic agents, dye spraying, and irrigation of the mucosal surface, all of which are time-consuming and complicated. In addition, the use of methylene blue carries the risk of oxidative DNA damage [15].

In contrast, the NBI system requires neither complicated preparation procedures nor dye spraying. Thus, magnifying NBI endoscopy was introduced for the diagnosis of atrophy and IM. Several classifications of gastric mucosal patterns seen with magnifying NBI endoscopy have been associated with the histological findings of atrophy and IM [7-9,16]. However, these classifications are complicated (4 to 6 types) and difficult to understand; this makes them difficult to implement in clinical practice. Therefore, more simplified approaches to the prediction of atrophy and IM are needed for use in clinical practice.

Uedo et al. first reported the use of LBC for the prediction of IM [10]. This study suggested that LBC, observed during magnifying NBI endoscopy, is a highly accurate predictor of IM, with a sensitivity, specificity, and accuracy of 89%, 93%, and 91%, respectively. The authors speculated that the LBC would be caused by differences in the reflectance of the light at the surface of the brush border. Similarly, the results of the current study demonstrate that LBC is a strong predictor of IM (sensitivity, 72.1%; specificity, 96.0%; accuracy, 84.9%).

However, many areas with a histological diagnosis of IM were not LBC positive. These findings led to a search for additional signs indicative of the presence of IM on magnifying NBI endoscopy. MTB was identified as another simple sign for the diagnosis of IM (accuracy, 81.7%). In addition, it was helpful in predicting atrophy (accuracy, 79.6%). Although the exact mechanism behind the occurrence of MTB remains unknown, it is likely that MTB is associated with changes in the gastric mucosa usually associated with atrophy and/or IM, such as the widening and shortening of the intervening part between foveolae.

In the present study, all LBC-positive areas were also MTB positive. In addition, the degree of IM increased with increasing MTB/LBC positivity, with moderate-to-severe IM more commonly seen in MTB^+^/LBC^+^ areas than in MTB^+^/LBC^-^ areas. On the basis of these findings, it is likely that MTB represents an early sign of IM as compared to LBC, with MTB observable in mild, followed by the appearance of LBC with progression to severe IM.

While endoscopic biopsy with subsequent histological evaluation is the current gold standard for the diagnosis of IM, the recently developed optical technology—magnifying NBI endoscopy—allows endoscopic visualization of regions of IM in the stomach without the need for biopsy. This method could also be used to increase the diagnostic yield in studies investigating the pathogenesis of other gastrointestinal diseases.

This study has several limitations. First, we focused only on the gastric body (the lesser curvature of the midbody and the greater curvature of the upper body). The findings of magnifying endoscopy are different between the gastric fundal and antral mucosa [7,8], and the regularly arranged ridges, which are seen in normal antral mucosa by magnifying endoscopy [17], may appear similar to MTB. Therefore, we chose to inspect only the gastric body, not the gastric antrum. Second, because the magnifying endoscopic findings were analyzed by only 1 experienced endoscopist in this study, interobserver reproducibility could not be evaluated. Although the reliability of some magnifying endoscopic findings has been reported recently [7,12], interobserver variability in the assessment of MTB and LBC needs to be evaluated before clinical application.

## Conclusion

In conclusion, MTB and LBC observed in the gastric mucosa with magnifying NBI endoscopy were highly accurate indicators for the presence of IM. MTB likely represents a sign of early gastric IM, while LBC appears with progression to severe IM. Further studies are necessary to assess interobserver variability for the detection of MTB and LBC.

## Competing interests

The authors declare that they have no competing interests.

## Authors’ contributions

Study concept and design - GAS, GHK, DYP, and JH; Acquisition of samples - GHK, DYP, and NRS; Analysis and interpretation of data - JKA, BEL, HYW, DYR, and DUK; Drafting of the manuscript - JKA, GHK, and DYP; Statistical analysis - JKA and GHK; Obtained funding - DYP; Co-senior author and study supervision - GAS. All authors read and approved the final manuscript.

## Pre-publication history

The pre-publication history for this paper can be accessed here:

http://www.biomedcentral.com/1471-230X/12/169/prepub
